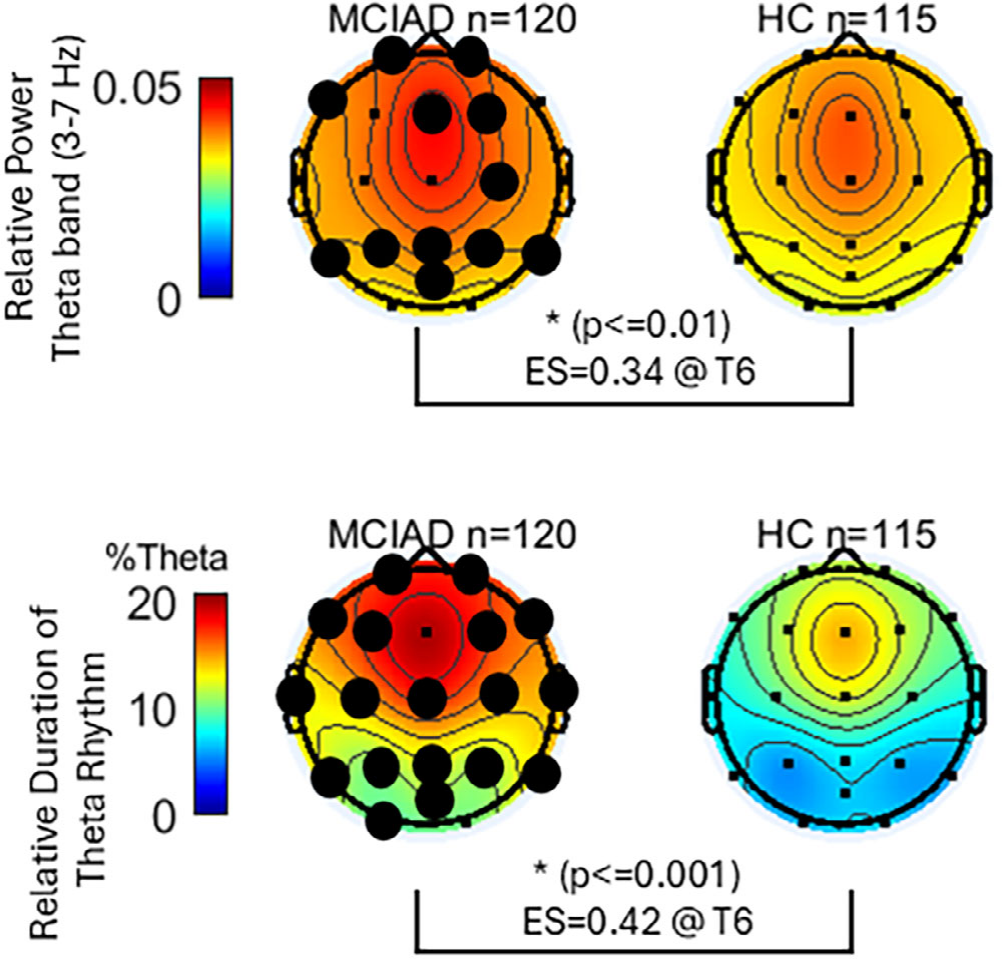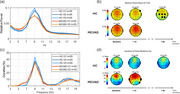# Resting state EEG Theta rhythms in healthy aging and Alzheimer's disease

**DOI:** 10.1002/alz70856_103986

**Published:** 2025-12-26

**Authors:** Amir H. Meghdadi, Caroline Ma, Ella J Thunen, Joanne M Hamilton, Brad F. Boeve, Erik K St. Louis, Corinne Fischer, David H Salat, Chris Berka

**Affiliations:** ^1^ Advanced Brain Monitoring Inc., Carlsbad, CA, USA; ^2^ Scripps Health, La Jolla, CA, USA; ^3^ Mayo Clinic, Rochester, MN, USA; ^4^ Department of Neurology, Mayo Clinic, Rochester, MN, USA; ^5^ Keenan Research Center for Biomedical Science, St. Michael's Hospital, Unity Health Network, Toronto, ON, Canada; ^6^ Massachusetts General Hospital, Boston, MA, USA; ^7^ Harvard Medical School, Boston, MA, USA; ^8^ Advanced Brain Monitoring, Inc., Carlsbad, CA, USA

## Abstract

**Background:**

Healthy aging and cognitive decline in Alzheimer's Disease (AD) and its precursor, Mild Cognitive Impairment (MCI), are linked to changes in resting‐state EEG power spectral density (PSD). Theta band (3‐7 Hz) power decreases with normal aging but increases with cognitive decline. However, frequency content does not always indicate true oscillatory activity. Oscillations reflect power concentration at a specific frequency, appearing as peaks in the frequency domain and visible rhythms in the time domain. Rhythmicity analysis quantifies oscillation persistence (percentage of time observed) independently of power. This study examined Theta power and Theta rhythms in healthy controls (HC) versus individuals with MCI/AD.

**Method:**

Participants included 32 AD, 88 MCI, and 115 and age‐ matched HC, who completed 5‐minutes of resting‐state eyes‐closed EEG at their baseline visit. PSD was computed for one‐second epochs. Relative Theta power was defined as the proportion of total power in the Theta band, averaging across epochs. Theta rhythm duration was defined as the percentage of epochs with Theta oscillations. Baseline differences and longitudinal changes were analyzed for a subset of participants: 28 MCI and 5 AD with at least one follow‐up visit, and 25 HC with at least two follow‐ups.

**Result:**

At baseline, the MCI/AD group exhibited significantly higher relative Theta power (*p* <0.01, df=233, ES=0.35 at T6) and a greater Theta rhythm duration (*p* <0.01, df=231, ES=0.41 at T6) compared to HC. Longitudinally, HC exhibited a significant reduction in Theta power by the second follow‐up (*p* <0.05, df=24, ES=0.51 at Cz), with no significant changes in Theta rhythms duration. In contrast, MCI/AD showed a significant increase in Theta rhythm duration (and not power) at first follow‐up visit (*p* < 0.05, df =32, ES=0.30 at T3).

**Conclusion:**

Theta power and Theta rhythm duration are both linked to cognitive decline in MCI and AD but differ from healthy aging patterns. While Theta power reflects age‐related changes, Theta rhythms duration may serve as a specific biomarker for disease progression in MCI and AD. These findings support the value of Rhythmicity analysis in EEG‐based biomarkers of cognitive decline.